# Strategies for Optimizing Acute Burn Wound Therapy: A Comprehensive Review

**DOI:** 10.3390/medicina61010128

**Published:** 2025-01-15

**Authors:** Andrei Cretu, Andreea Grosu-Bularda, Eliza-Maria Bordeanu-Diaconescu, Florin-Vlad Hodea, Vladut-Alin Ratoiu, Catalina-Stefania Dumitru, Mihaela-Cristina Andrei, Tiberiu-Paul Neagu, Ioan Lascar, Cristian-Sorin Hariga

**Affiliations:** 1 Department 11, Discipline Plastic and Reconstructive Surgery, “Carol Davila” University of Medicine and Pharmacy, 050474 Bucharest, Romania; andrei.cretu@drd.umfcd.ro (A.C.);; 2 Clinic of Plastic Surgery and Reconstructive Microsurgery, Clinical Emergency Hospital of Bucharest, 014461 Bucharest, Romania

**Keywords:** burn wound, wound healing, imaging techniques, therapeutic strategies, novel therapies, skin substitutes

## Abstract

Recent advancements in acute burn wound therapy are transforming the management of burn injuries, with a focus on improving healing times, graft integration, and minimizing complications. However, current clinical treatments face significant challenges, including the difficulty of accurately assessing wound depth and tissue viability, which can lead to suboptimal treatment planning. Traditional closure methods often struggle with issues such as delayed wound closure, limited graft survival, inadequate tissue regeneration, and insufficient vascularization. Furthermore, managing infection and minimizing scarring remain persistent obstacles, impacting functional recovery and aesthetic outcomes. Key areas of innovation include advanced imaging techniques that enable more precise assessment of wound depth, size, and tissue viability, allowing for more accurate treatment planning. In addition, new closure strategies are being developed to accelerate wound closure, enhance graft survival, and address challenges such as tissue regeneration, vascularization, and infection prevention. These strategies aim to optimize both functional recovery and aesthetic outcomes, reducing scarring and improving the quality of life for burn patients. While promising, these emerging techniques require further research and clinical validation to refine their effectiveness and expand their accessibility. Together, these innovations represent a significant shift in acute burn care, offering the potential for more personalized, efficient, and effective treatments.

## 1. Introduction

Globally, burns rank as the fourth most frequent cause of accidental injury, following traffic accidents, falls, and drownings, leading to the need for medical care for millions of patients annually. Burn injuries lead to intricate biological effects, triggering a series of complex processes, such as immune and inflammatory responses, metabolic imbalances, and burn-induced coagulopathy. Therapeutic strategies for severely burned patients involve a combination of critical care treatment and targeted wound management, supported by a multidisciplinary team. Despite significant advancements in burn treatment, the complexity of these injuries continues to present challenges in managing both systemic and wound-specific complications [1,2,3,4].

It is crucial to fully grasp the complexities of the various mechanisms driving microvascular dysfunction when deciding upon treatment strategies for burn wounds. The literature typically highlights three key categories: vessel thrombosis caused by vascular damage, increased production of inflammatory mediators, and the presence of pro-apoptotic factors [5,6]. Moreover, severe burns are characterized by a general systemic response, called systemic inflammatory response syndrome (SIRS), involving the modification of a range of cellular mediators, cytokines, pro-inflammatory molecules, and adipokines. SIRS often leads to multiple organ dysfunction syndrome, requiring a multidisciplinary approach in order to support vital organs and keep biological parameters within acceptable values [5,7,8]. This unfavorable evolution can be monitored through the modification of various biochemical parameters, with recent studies suggesting that less commonly used markers, such as plasminogen activator inhibitor-1 (PAI-1) or platelet factor 4 (PF4), might be useful. PAI-1 and PF4 generate a procoagulant effect, with an upward trend in the 48 h and 24 h post-burn, respectively, increasing the already high risk of deep venous thrombosis and pulmonary embolism [6,9,10,11,12].

Effective management of burn wounds is essential to improving outcomes and minimizing the risk of wound conversion, where initially superficial burns deepen over time. This conversion process is determined by various factors, including delayed treatment, inadequate blood flow, as well as tissue damage from inflammation. Strategies to mitigate this progression focus on early and appropriate interventions that stabilize the wound environment, limit infection, and promote healing. Therapeutic approaches include optimizing wound debridement, applying topical agents to reduce bacterial load, and using advanced technologies such as biologic and skin substitutes to enhance tissue regeneration. Addressing the multifaceted nature of burn injuries requires a comprehensive and individualized treatment plan that integrates these modalities effectively [13].

Burn victims are particularly vulnerable to infections, especially those caused by drug-resistant bacteria. These infections often lead to prolonged hospitalizations, slower wound healing, increased treatment costs, and higher mortality rates. Sepsis and multiorgan failure are the leading causes of death following severe burns, making infection prevention and management a critical aspect of burn care. Among the most frequently encountered bacteria, some are multidrug-resistant, extensively drug-resistant, or even pan-drug-resistant. The Infectious Disease Society of America has identified six bacteria, known as “ESKAPE pathogens” (Enterococcus faecalis, Staphylococcus aureus, Klebsiella pneumoniae, Acinetobacter baumannii, Pseudomonas aeruginosa, and Enterobacter spp.) that pose significant therapeutic challenges due to their growing resistance to antibiotics, even those considered last-line defenses. Early excision and autologous skin grafting are considered the gold standard for managing full-thickness and deep partial-thickness burns with expected healing exceeding 3 weeks, or placed in functional areas, where vicious scarring represents a significant problem. Wounds that fail to heal within three weeks, such as deep burns, experience prolonged inflammation and delayed re-epithelialization, which disrupt the normal healing process. This leads to excessive collagen production, impaired angiogenesis, and an altered extracellular matrix, all of which contribute to the increased risk of scarring. Thus, this approach is particularly effective because it reduces the risk of infection, promotes wound healing, and minimizes scarring. Debridement is mandatory, since removing burn eschar eliminates a major source of inflammation and a fertile environment for bacterial growth [14,15].

For burn trauma survivors, the aftermath is often severe. According to a WHO report on child injury prevention, 8% of survivors are left with permanent physical disabilities, with the most frequent long-term effects including hypertrophic scars, keloids, contractures, and in some cases, the need for amputation. Beyond physical disabilities, burns have a significant emotional impact on survivors. The psychological effects depend not only on the characteristics of the burn but also, and more importantly, on factors like family support, mental health, and individual coping mechanisms. In high-income countries, patients from disadvantaged family situations may face challenges in returning to school or community life. In contrast, in low- and middle-income countries, survivors often struggle with social reintegration, face family abandonment, and encounter difficulties finding employment, which can lead to poverty [16,17].

The aim of this narrative review is to summarize recent advancements in burn wound diagnosis, treatment modalities, and their potential to enhance patients’ outcomes.

## 2. General Principles in Burn Wound Treatment

The pathophysiological response of burns at the skin level has different severity grades, with the extent of tissue damage depending on factors such as temperature, exposure duration, and the specific heat of the traumatic agent. In 1953, Dr. Douglas Jackson introduced a model of burn injury that describes three concentric zones of damage, which continues to be widely recognized today. The zone of coagulation is the most affected, where all cells are non-viable, and the extracellular matrix proteins are denatured due to direct contact with the burn-causing agent. The focus in this zone is on debridement and infection control, as the damaged cells cannot be restored. Surrounding this is the zone of stasis, which contains initially viable tissue which, however, suffers from hypoperfusion due to vasoconstriction. While recovery in this zone is possible, it may progress to necrosis within 24–48 h if hypoperfusion persists due to inadequate fluid resuscitation or in the presence of edema or infection. Factors such as advanced age, smoking, diabetes, and other chronic conditions can worsen this progression. Given the irreversibility of the coagulation zone, treatment aims to prevent the stasis zone from deteriorating into coagulative necrosis. The outermost zone, known as the zone of hyperemia, consists of viable cells and associated vasodilation due to inflammation and can fully recover within 7–10 days, provided hypoperfusion or infection is avoided [18,19,20].

Burns are classified by depth corresponding to the skin layers. First-degree burns (superficial) are those affecting only the epidermis. These are red, painful, and heal on their own within 3–4 days, leaving no scars, such as those provoked by sunburn. Second-degree burns are subdivided into superficial partial-thickness and deep partial-thickness. Superficial partial-thickness (IIA degree) burns affect the epidermis and the papillary dermis and are red and painful with clear blisters. They heal in about 14 days, with minimal scarring. Deep partial-thickness burns (IIB degree) are those burns that penetrate deeper into the reticular dermis. They are pale with a spotted appearance, causing discomfort rather than pain, and may take up to 3 weeks to heal, but not through re-epithelization but rather by wound contracture, which is why surgical intervention is often preferred to prevent scarring and contractures. Third-degree or full-thickness burns affect all skin layers including the hypodermis. The skin appears white or charred, and there is no pain due to nerve damage. Healing is impossible without skin grafting. Lastly, fourth-degree burns, which are sometimes described in the literature, extend beyond the skin to muscle and bone. They often require extensive debridement, reconstruction, or even amputation [20,21,22,23].

### Initial Response in the First 48–72 h

The first maneuver on a burn patient, not only at the site of the accident, but also on arrival in the emergency room of the hospital, is to check the vitals following the ABCDE guidelines: airway, breathing, circulation, disability, and exposure. Only after thoroughly resolving any issues regarding the patient’s vital signs can the medical team pursue the next steps in the management of the burn patient [18].

All patients should be washed with antiseptic and antimicrobial solutions, undergo an initial mechanical debridement in the burn center, and then, some of them will require emergency surgery immediately after admission, usually consisting of decompression incisions. That is most frequently the case for circumferential or near-circumferential full-thickness burns, where the formation of the eschar leads to a decrease in skin distensibility, which is exacerbated by the normal edematous reaction of the tissues and, furthermore, by the necessary fluid resuscitation. Thus, the pressure rises inside the soft tissues, leading to compartment syndrome in the extremities, respiratory restriction if the thorax or abdomen is involved, or even intraocular pressure in patients with periorbital burns. The medical team should assess the patient and establish the need for immediate surgery as soon as possible, bearing in mind that the clinical signs of compartment syndrome might be delayed, so the indication for escharotomy can be extended prophylactically in patients who need massive fluid resuscitation [24].

In most traumatic injuries, the classic signs of compartment syndrome (pain, pallor, pulselessness, paresthesia, paralysis) indicate the need for a fasciotomy. However, in thermal burns, the full-thickness inelastic eschar is the one that restricts the tissue expansion and not the fascia. Hence, fasciotomies are relatively unusual for thermal burns, but they must be considered in cases of delayed treatment. On the other hand, fasciotomies are the standard of care in electrical burns, where the damage caused by the high voltage affects the muscles. Early fasciotomies have been shown to decrease the need for limb amputations [24,25]. However, amputations can be lifesaving for the burn patient, and it has been shown that the earlier the amputation is performed, the lower the level of the amputation. The suggested window for evaluating muscle viability is 3–5 days after injury and, should the muscles be found to be no longer viable, the prior fasciotomy and the following early amputation might spare a major articulation [25,26,27].

## 3. Emerging Techniques in Burn Therapy

### 3.1. Burn Wound Assessment

**Figure 1:**
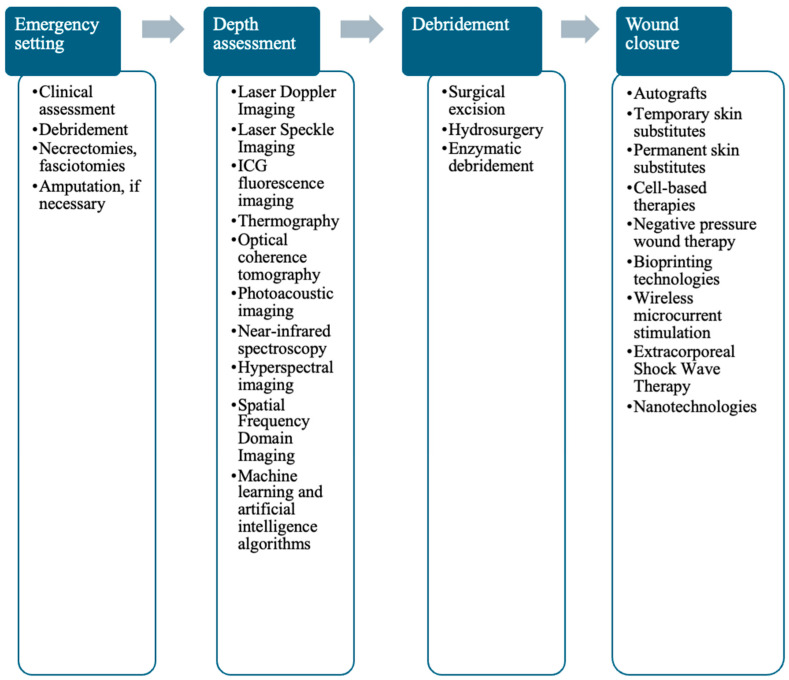
Chronological algorithm for burn wound treatment.

The depth of the burn wound must be accurately assessed, since its healing potential is directly dependent on the depth of the lesion, which will guide the appropriate therapeutic strategy. Clinical examination was for a long time the only method for assessing burn wounds. However, despite its value, clinical examination has limitations, such as subjectivity and dependence on the experience and expertise of the examiner, which has led to the development and use of additional methods and modern technologies. Clinical evaluation is the first pillar of the assessment, but imaging technologies have recently advanced the assessment potential of the burn wound, improving the ability to non-invasively evaluate burns and closely monitor the healing progress [28]. Figure 1 shows the chronology of burn wound treatment and the available and potential technologies that can be used.

#### 3.1.1. Laser Doppler Imaging (LDI)

**Figure 2:**
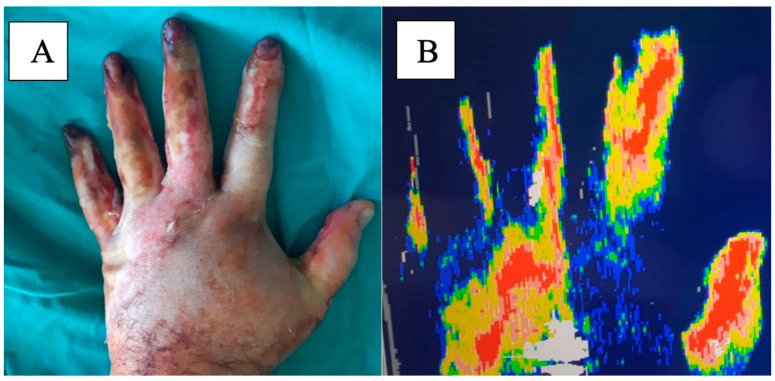
Laser Doppler Imaging in a hand burn. (**A**) Clinical aspect. (**B**) LDI imaging.

Laser Doppler Imaging (LDI) is an advanced, non-invasive diagnostic tool, introduced by Essex and Bryne in 1991 [29], used in burn care to assess skin perfusion and determine burn depth. It works by directing a low-power laser beam onto the burn site, where the light is scattered by moving red blood cells in the microcirculation, producing a Doppler shift. This shift is used to generate real-time images that map out blood flow, which is crucial for assessing tissue viability. Studies have shown that LDI can differentiate between superficial partial-thickness burns, which exhibit increased blood flow, and deeper burns, such as full-thickness burns, where blood flow is compromised, as shown in Figure 2. As a result, LDI is particularly useful for clinicians in making critical decisions regarding the need for excision or grafting, and it offers the potential to predict wound healing (time to complete re-epithelization) by monitoring perfusion levels during the early stages of burn injury. Additionally, it proves particularly beneficial in cases where clinical judgment may be limited, such as in patients with obscured injuries or those with dark skin tones, where other assessment methods might fall short [30,31].

However, while LDI shows great promise, its use is not without limitations. For instance, its accuracy may be influenced by the stage of the burn and the underlying condition of the tissue, which could affect the perfusion readings. Furthermore, LDI technology requires significant expertise to interpret the results accurately, and it is not as widely available as other methods. Nonetheless, ongoing improvements in LDI technology and its integration with other diagnostic tools, such as hyperspectral imaging, may help to address these challenges [30,32].

Mirdell et al. proved that two consecutive measurements, the first one in the first 24 h post-injury and the second one between 72 and 96 h post-injury, can reach 100% specificity and sensibility in predicting the prognosis of the burn wound, compared to only 67% for clinical evaluation [31]. Several other studies have shown similar results, with LDI accuracy in the range of 90–100% and clinical accuracy between 50–75% [33].

However, the device is costly, bulky, difficult to move, and requires significant time to use. Furthermore, patients must remain still during measurements to prevent artifacts, which can be especially difficult when assessing burn wounds in children [34]. A faster LDI, called the Laser Doppler Line Scanner (LDLS), was found to be as accurate as the classic LDI. Compared to normal LDI, that can scan as much as 2500 cm^2^ in 2 min, LDLS covers 300 cm^2^ in only 4 s, enabling easier use, even in an outpatient setting [35].

#### 3.1.2. Laser Speckle Imaging (LSI)

Laser Speckle Imaging (LSI), first used in the 1980s using analog techniques for biomedical use [36], assesses burn depth by interpreting blood flow fluctuations in tissue illuminated by a laser. It uses changes in the speckle pattern to generate perfusion maps, correlating blood flow with burn severity. Studies show LSI accurately predicts healing times and demonstrates high sensitivity and specificity, even in varied clinical settings. Compared to Laser Doppler Imaging (LDI), LSI offers faster image capture, higher resolution, and affordability. However, both methods face limitations due to dynamic tissue changes and may not fully reflect actual blood perfusion [37].

#### 3.1.3. Indocyanine Green (ICG) Fluorescence Imaging

Indocyanine Green (ICG) fluorescence imaging is an emerging technique that enhances the visualization of blood flow in burn wounds. Fluorescein fluoresce was first used to assess burn wound depth in 1943 [38]. With a spectral absorption of 800 nm, ICG can be detected under near-infrared light, which is used to excite the dye, making areas with good perfusion glow brightly. The dye is injected intravenously, where it binds to its transporter through the circulatory system, albumin. ICG imaging helps assess the viability of burn tissue and can be used intraoperatively to guide surgical debridement and grafting. It provides real-time feedback, allowing surgeons to assess tissue perfusion immediately during surgery [39]. A multicentric, triple-blind study that compared the results of clinical evaluation and those provided by ICG imaging found a 100% specificity and sensibility for the latter [40].

Superficial partial-thickness burns exhibit fast uptake and appear bright and homogenous, as the vascularization is still intact, while deep partial-thickness burns appear darker, with a mottled fluorescence and show slower uptake, since the dermal plexus is partially destroyed. Lastly, full-thickness burns will exhibit fluorescence only in large vessels, since the entire dermal plexus is damaged [41].

#### 3.1.4. Thermography

Thermography, first introduced in 1961, uses infrared radiation to create heat maps of the skin, detecting subtle changes in temperature that correspond to blood flow and tissue viability. In burn care, thermography helps assess tissue perfusion, identifying areas of deep burn or ischemia [42,43].

**Figure 3:**
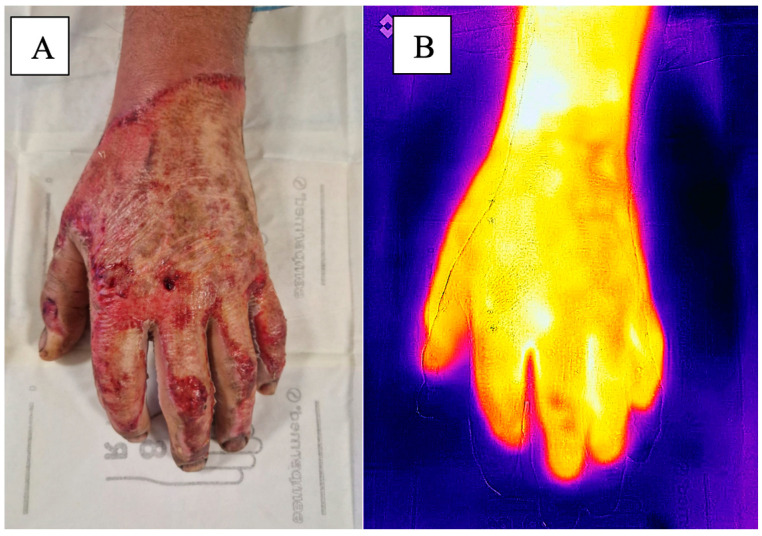
(**A**) Dorsal side of a burned hand with alternating areas of partial-thickness and full-thickness burn wounds. (**B**) Thermography imaging of the same hand.

Static infrared imaging determines burn depth by identifying temperature variations—full-thickness burns appear cooler than healthy skin or superficial burns because blood vessels are damaged in these areas (as shown in Figure 3). In contrast, active infrared imaging applies cold stimulation before observation to measure the recovery time to normal temperatures: superficial burns recover more quickly than full-thickness burns. Both active and static imaging are quick, noninvasive methods that provide real-time results within minutes. Their accuracy surpasses that of clinical evaluations, with active imaging achieving an accuracy rate of 83% [43].

Unlike LDI, thermal imagers are affordable, user-friendly, compact, and can easily connect to mobile phones and tablets, capturing images within seconds. These devices can be especially useful in situations with limited consultation time or when LDI is unavailable or impractical [44].

#### 3.1.5. Other Methods

Optical coherence tomography (OCT) has been successfully utilized for characterizing burn injuries. OCT detects changes in the polarization of light reflected from burn tissue, enabling measurements that aid in assessing tissue structure and function, ultimately helping with the evaluation of wound depth. A reduction in collagen birefringence is thought to correlate with increased burn depth. However, OCT cannot reliably assess superficial burns in the epidermis due to its limited resolution, which is insufficient for visualizing individual cells and accurately detecting tissue damage, and it does not effectively measure skin perfusion [45,46]. OCT-based angiography (OCTA) is an advanced version of OCT that visualizes functional microvascular networks below the tissue surface. Unlike earlier versions of OCT, OCTA can capture and measure functional blood flow, fluid accumulation, and structural details all within a single scan [47].

Photoacoustic imaging (PAI) is a promising biomedical imaging technology, first described by Alexander Graham Bell as a conversion of optical energy to audible pressure waves [48], with greater penetration depth than optical imaging. With its high contrast and deep penetration, PAI has shown value for imaging deeper tissues. PA signals correlate directly with blood perfusion, which decreases in burned tissue due to blood vessel occlusion. However, this approach only accounts for blood distribution’s effect on PA signal intensity, which does not accurately represent actual blood flow. Additionally, because PAI relies on light absorption, it cannot provide information on light scattering, which is important for assessing the skin’s scattering structure and identifying the extent of the burn wound [49,50].

Near-infrared spectroscopy is a non-invasive technique used to monitor hemodynamic changes by measuring concentrations of oxyhemoglobin and deoxyhemoglobin in the blood, based on their capacity to reflect light within the near-infrared range (700–900 nm), where most tissues are relatively transparent [51]. NIRS demonstrated a significant increase in oxygen saturation and total hemoglobin in superficial burn wounds compared to control areas, while full-thickness burns exhibited a reduction in oxygenation and total hemoglobin levels. Moreover, local oxygen saturation positively correlates with blood flow, as measured by LDI [52,53].

Hyperspectral imaging (HSI) is a cutting-edge technology that captures a wide range of spectral data from burn wounds, providing valuable insights into tissue characteristics such as oxygenation, blood perfusion, and hemoglobin levels. The first study describing burn wounds through the use of HIS was published in 2015. By analyzing the spectral signatures of the burn site, HSI allows for precise differentiation between various burn depths, including superficial and deep injuries. Studies have shown that HSI can detect changes in the optical properties of burned tissues, allowing clinicians to assess burn severity and predict healing potential with greater accuracy compared to traditional visual inspection methods [54]. The clinical application of HSI has expanded in recent years, particularly for assessing burns in specific anatomical areas, such as the hands and upper extremities. HSI improves the early identification of areas that may require surgical intervention, such as excision and grafting, by accurately determining the extent of ischemia and tissue damage. In addition to burn severity assessment, HSI has shown potential in predicting wound healing outcomes, offering a significant advantage in treatment planning. HSI allows clinicians to assess the viability of burn areas and predict which regions will likely heal naturally versus those that may require more intensive treatments, aiding in the evaluation of healing progression and assisting in the early detection of complications, such as infection or delayed healing [55,56].

Spatial Frequency Domain Imaging (SFDI) is an emerging, non-invasive optical technique used for assessing burn wounds by analyzing tissue properties such as oxygenation, perfusion, and depth. SFDI uses modulated light to penetrate tissue and measures the variations in the light’s interaction with tissue at different spatial frequencies. This technique offers high spatial and temporal resolution, allowing clinicians to precisely assess burn depth, monitor healing, and identify areas requiring surgical intervention, such as excision or grafting. Recent studies have demonstrated SFDI’s ability to classify burn severity and predict healing outcomes, providing a more objective, real-time assessment compared to traditional visual inspection methods [57,58]. In addition to its ability to assess burn wounds, SFDI has shown promise for monitoring skin graft healing. By providing detailed images of tissue perfusion and oxygenation, SFDI aids clinicians in evaluating the viability of grafts and detecting early signs of complications like ischemia or infection [59]. Furthermore, the integration of machine learning with SFDI data, as demonstrated in various studies, allows for the development of predictive models that can improve the accuracy of burn classification and guide treatment decisions [58].

In recent years, technological advancements have pushed forward the integration of new technologies into medicine, including in the assessment of burn depth. Multiple studies have successfully demonstrated the use of various machine learning (ML) algorithms that autonomously analyze optical parameters from imaging methods to predict burn depth with accuracies up to 92.5%, which is close to that of histopathological evaluation [60,61,62,63]. As expected, each study uses different imaging techniques, such as ultrasound imaging, OCT, NIRS, or digital photography. The most frequent ML algorithms in these studies are the support vector machine (SVM) and convolutional neural network (CNN) [63]. Additionally, artificial intelligence (AI) systems have been developed to estimate injury depth, achieving accuracy rates of up to 80–95%, which can prove more than useful in settings where there are no specialized burn centers in the proximity. AI could outperform traditional clinical methods in predicting burn depth and healing outcomes. For instance, burn depth predictions using AI algorithms like neural networks were found to improve significantly when dealing with multiclass categorization problems, mapping them onto simpler binary classifications [64,65,66]. AI studies showed good efficacy in estimating burn depth even when the data were provided in the form of mobile-captured images with potential background noise, showing strong correlations with LDI-based assessments and providing an accessible and efficient alternative for burn evaluation, especially useful in situations with limited resources [67]. However, there is some variability in AI model performance, particularly related to dataset diversity and quality, which can impact its reliability. Additionally, the integration of AI into real-world clinical practice still requires overcoming hurdles such as clinician trust and regulatory approval [65]. AI systems are expected to evolve toward providing more precise, individualized assessments by integrating multiple data sources, such as medical imaging, clinical history, and patient-specific factors, including the integration of AI with real-time, non-invasive diagnostic tools. Future directions will likely focus on improving the ability to predict burn healing trajectories, including determining whether a wound will heal through secondary intention or require surgical intervention (e.g., excision or grafting). These predictions could be made with increasing accuracy, using advanced machine learning algorithms and deeper integration of temporal data to track healing progress over time [67,68].

### 3.2. Burn Wound Debridement

#### 3.2.1. Early Surgical Excision

Since the burn eschar is prone to infection and promotes the hypermetabolic state of the patient, the standard of care of burn wounds is early excision, which reduces mortality and hospital length of stay, followed by grafting using autografts [69,70,71].

Tangential excision is the most used method for debriding burn wounds. This technique involves gradually removing tissue layers until reaching healthy tissue suitable for grafting, which can be identified either by pinpoint bleeding at the dermal level or by the presence of adequately vascularized adipose tissue. Although it may result in more significant bleeding, tangential excision offers superior aesthetic and functional outcomes compared to fascial excision [69,72].

Fascial excision involves removing all of the affected tissue down to the fascia, using electrocautery. This technique is employed for full-thickness burns to prevent massive bleeding and to better control infection, if present, despite drawbacks such as lymphedema and a less aesthetic outcome [71].

#### 3.2.2. Hydrosurgical Excision

An alternative approach for debriding burn injuries is the Versajet^®^ hydrosurgical system, which uses a high-pressure saline jet that acts like a scalpel, based on the Venturi effect, both removing and suctioning necrotic tissue from the wound at the same time [73].

This technique offers superior precision and control compared to traditional excision, and it has been shown to be safe, provide effective debridement, and preserve viable tissues, making it suitable even for sensitive areas such as the hands or face. The Versajet^®^ system is preferred for partial-thickness burns, as it is less effective for debriding full-thickness burns. However, these same characteristics make it less advantageous for large areas that require rapid debridement [69,73].

Hydrosurgical excision combined with skin grafting decreases intraoperative blood loss per unit area of grafted skin, improves scar quality one year after the injury, does not raise treatment costs per unit of burned area, and has similar complication rates with normal surgical excision [74,75].

#### 3.2.3. Enzymatic Debridement

As the name suggests, this debridement technique uses proteolytic enzymes to break down burned and devitalized tissue without affecting healthy tissue. The enzymatic debridement process involves initially cleaning away all non-viable tissue debris, applying saline and chlorhexidine to the burned areas, followed by the enzymatic product for 4 h, after which it is removed, and cleaning the wounds again with saline. Advantages include faster re-epithelialization, reduced healing time, fewer required surgical excisions, decreased need for autografting, and minimized bleeding. However, enzymatic debridement can cause local erythema and significantly increased pain, especially during application and removal of the product, which can be counteracted with sufficient analgesia [76,77,78].

The European consensus on bromelain-based enzymatic debridement (Nexobrid^®^), derived from the stem of the pineapple plant, indicates that prior application of silver sulfadiazine is not recommended, due to interference with the enzymatic activity. This technique can be used on a maximum burn surface area of 15% TBSA in a single session. For circumferential burns, enzymatic debridement can be applied early on, potentially preventing the need for escharotomies, though not for fasciotomies. Enzymatic debridement is particularly beneficial in patients who are not candidates for surgery due to medical comorbidities or in cases where donor sites are limited [79].

**Figure 4:**
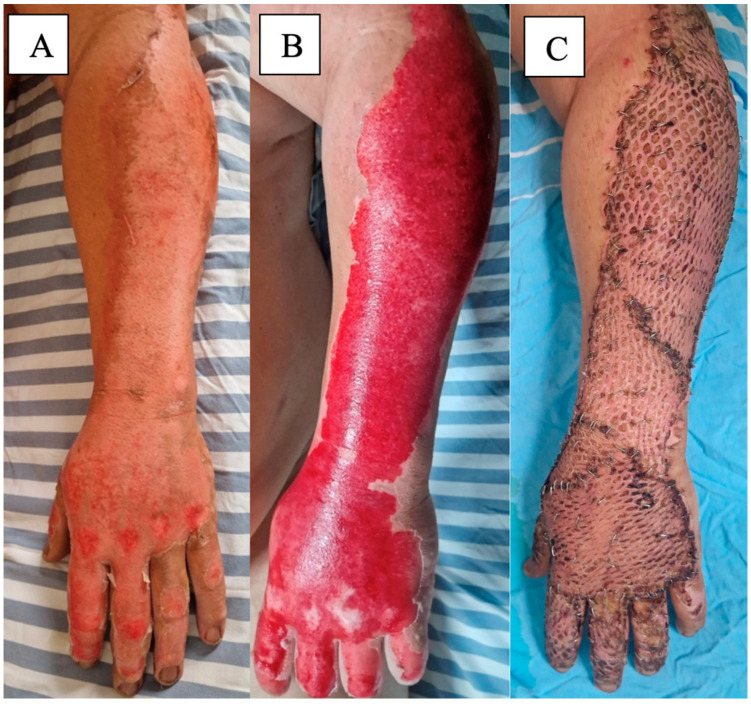
(**A**) Partial-thickness burn wound on the dorsal side of the forearm and hand. (**B**) Aspect after enzymatic debridement using Nexobrid^®^. (**C**) Final step of treatment, after applying split-thickness skin grafts.

Enzymatic debridement (see Figure 4) offers several benefits, including reduced surgical trauma, faster wound bed preparation, and preservation of viable tissue. However, it may not be suitable for all burn wounds, especially those with extensive or deep eschar. In some cases, the use of enzymatic debridement may still need to be followed by surgical intervention if complete debridement is not achieved [73,76,79].

Surgical excision, hydrosurgical excision, and enzymatic debridement are distinct approaches to burn wound management, each with specific strengths and limitations. Surgical excision, the traditional gold standard, is particularly effective for full-thickness and extensive burns, offering complete and immediate removal of necrotic tissue. In contrast, hydrosurgical excision provides a more precise and controlled method of debridement, preserving healthy tissue. It is particularly effective for partial-thickness burns and sensitive areas such as the face or hands. However, it is less efficient for large wounds requiring rapid debridement and for full-thickness burns, where surgical excision remains superior. Enzymatic debridement, distinct from the mechanical approaches of surgical and hydrosurgical methods, removes necrotic tissue while sparing healthy tissue. This method is less invasive, reducing surgical trauma and preserving the wound bed. Unlike surgical and hydrosurgical excision, enzymatic debridement does not require specialized equipment or a surgical theater but can be slower in addressing extensive wounds. Pain during application and local erythema are significant drawbacks. Hydrosurgical excision and enzymatic debridement both offer greater precision and tissue preservation compared to surgical excision, but their applications differ. Hydrosurgical systems are ideal for smaller, well-defined wounds and areas requiring detailed debridement, whereas enzymatic debridement excels in non-surgical candidates or where donor sites are limited. Despite their advantages, neither can match the speed and thoroughness of surgical excision for managing extensive or deep burns, where immediate preparation for grafting is essential. Overall, surgical excision provides the most definitive treatment for extensive wounds, while hydrosurgical excision and enzymatic debridement cater to specific needs, emphasizing precision, preservation, and reduced invasiveness. The choice between them depends on burn depth, extent, patient condition, and treatment goals, with their strategic use often enhancing overall outcomes.

### 3.3. Burn Wound Closure

#### 3.3.1. Skin Autografts

Skin autografts are divided into split-thickness and full-thickness grafts, depending on the harvested skin layers. The use of autografts becomes more challenging as burn wounds increase in size.

Full-thickness grafts, containing both epidermis and dermis, provide superior elasticity, flexibility, and texture, offering better aesthetic results and scarring. However, their viability may be compromised due to the dermal thickness during revascularization, and the donor site often requires primary closure due to dermis removal, with limited anatomical donor areas (e.g., supraclavicular, retroauricular, inguinal). Full-thickness grafts are typically used for facial and hand defects [69,73,80].

For larger burn areas, split-thickness skin grafts, which include the epidermis and part of the dermis, are more common. The donor area can be reused after healing, though repeated harvests may lead to hypopigmentation and increased contracture risk. For small lesions, unexpanded grafts yield better cosmetic outcomes but carry risks of seromas or hematomas [69,73,80].

Instruments like manual dermatomes and modern rotary dermatomes are commonly used for harvesting STSGs. Rotary dermatomes are especially helpful in treating large burns because they offer speed and precision, while manual instruments like the Weck guarded Goulian knives and Watson knives are great for smaller or delicate areas, providing excellent control for harvesting thin skin layers or removing dead tissue [81].

Expanding grafts is often necessary for large defects. A 2:1 expansion facilitates application and drainage but yields less aesthetic results due to perforations. Larger expansions, such as 4:1, cover extensive areas, often necessitating a sandwich technique with an overlaid allograft. For critical burn cases, even higher ratios may be used, though these are unsuitable for visible areas like the face and hands, where unexpanded grafts are preferable [82].

The Meek micrografting technique divides a graft into small square fragments, achieving up to 9:1 expansion for coverage of extensive areas (up to 75% of total skin surface), with better viability due to lower metabolic demands and fewer interventions needed. Donor sites should ideally match the recipient area in pigmentation, with common donor areas including the thigh, hip, and gluteal region for easier harvesting and scar concealment [83]. Traditional meshing devices create slits in the skin graft, allowing it to stretch and cover a larger area. However, Meek grafting offers greater uniformity and can achieve higher expansion ratios, making it particularly effective for extensive burns. These advancements in tools and techniques highlight the innovative methods being used in modern burn and wound care [84].

#### 3.3.2. Temporary Skin Substitutes

Allografts, derived from cadaveric skin, are biologic dressings that only temporarily cover the wound before it is ready for closure. They can revascularize when placed on an excised burn wound but are eventually rejected after 3–4 weeks due to their non-self-antigenic properties. They serve as temporary coverage without needing immunosuppressants and prevent the desiccation of tissues. Moreover, allografts help the surgeon to evaluate the suitability for later autografting [85].

Xenografts, primarily porcine and bovine xenografts, provide temporary wound coverage, reduce pain, and protect against infection, though they do not vascularize, but only adhere to the wound bed. Thus, they are mainly used for partial-thickness burns and donor sites [86,87]. Recently, newer fish-based xenografts have caught more attention because of their capacity to offer anti-inflammatory benefits and higher porosity, supporting fibroblast activity and resisting bacterial invasion for short periods [88].

The human amniotic membrane (HAM) is a valuable option for temporary wound coverage, especially for burns and trauma on irregular surfaces like the face. Its multi-layered structure consists of an epithelium, which contains pluripotent stem cells, a basal membrane rich in extracellular matrix proteins like collagen and fibronectin, and additional stromal layers containing regenerative molecules. HAM provides essential wound healing benefits, including immunomodulatory effects, anti-scarring, antimicrobial properties, and tissue regeneration. Its flexibility makes it suitable for one-time application, potentially reducing pain and increasing comfort for patients [89,90]. Despite these advantages, it presents challenges due to storage requirements, immediate availability, and risks of disease transmission. To address these, human acellular amniotic membrane (HAAM) was developed, utilizing infrared and microwave irradiation drying and gamma-ray sterilization, making it easier to store and handle while removing viable cells to minimize immune response [91,92].

Synthetic skin substitutes (see Table 1) provide sterile, consistent wound coverage, absorbing exudates and preventing infection when carefully monitored, but also maintain an appropriately moist environment for healing. They can be polyurethane-based dressings, silicone-based dressings, hydrocolloid dressings, hydrofiber dressings, alginate dressings or based on other synthetic materials (polylactic acid membranes or polyester mesh impregnated with petroleum jelly, etc.) [93,94,95].

#### 3.3.3. Permanent Skin Substitutes

Ideally, the best solution for covering burn wounds would be a product that mimics skin properties while remaining integrated during healing. Most of the available substitutes mainly mimic dermal elasticity and require subsequent split-thickness skin grafting [98,99].

Acellular Dermal Matrices (ADMs) are scaffolds that promote tissue regeneration by providing a structure for cell growth and integration. Used in both acute and reconstructive burn care, ADMs are valuable for cases where autografts are not feasible, especially in full-thickness burns. Combined with thin split-thickness grafts, ADMs have shown improved functional and cosmetic results, reduced scar contracture, and enhanced graft integration [69,73].

AlloDerm^®^ is an allogenic dermis derived from decellularized and de-epithelialized human cadaver skin, by undergoing freeze-drying to eliminate antigenicity and prevent rejection. Since it lacks an epidermal barrier, it must be paired with a thin epithelial autograft when applied on the wound bed [98,100].

Integra^®^ consists of a temporary silicone epidermal layer and a bovine collagen–chondroitin matrix for permanent dermal replacement, allowing fibroblast infiltration and revascularization. After 2–3 weeks, once the dermal matrix is integrated, the silicone layer can be replaced with an autograft. The epidermal layer serves as a barrier against infection and dehydration, while the dermal layer promotes the synthesis of a new dermal layer. In severe burns, Integra^®^ can integrate over poorly vascularized areas like tendons, where skin grafts would not adhere. Histological studies show Integra^®^ resembles natural skin closely, with better elasticity [98,99,101].

While Integra^®^ requires a two-step process, other dermal substitutes allow for a single-step procedure. Matriderm^®^, the first to do this, consists of a freeze-dried scaffold of bovine collagen and elastin, applied directly to the wound bed or after wetting with saline. Clinical studies note that graft integration can be slower, likely due to the interposition of the scaffold between the graft and the wound bed, but, on the other hand, scar quality proves to be superior [98,99,102].

Novosorb^®^ Biodegradable Temporizing Matrix is a synthetic, acellular dermal substitute used in the management of burns, trauma wounds, and other complex skin defects. Unlike biologic substitutes like AlloDerm and Integra, BTM is fully synthetic, composed of a porous polyurethane foam matrix topped with a temporary sealing layer. The matrix serves as a scaffold for cellular infiltration and vascularization, promoting dermal regeneration, while the sealing layer protects the wound during the healing process. Once the new dermal layer forms, the sealing layer is removed, and a split-thickness skin graft is applied. BTM offers advantages such as a long shelf life, resistance to immunogenic reactions, and ease of use, making it a valuable tool in modern burn care and reconstructive surgery [103].

An ideal skin replacement method would replace both dermal and epidermal layers simultaneously. A logical approach involves combining epithelial cells with a dermal scaffold in the lab. Several research teams have developed composite skin substitutes, such as Orcel^®^, a bilayered substitute made of fibroblasts and keratinocytes, but its allogenic cells do not survive long in the wound bed [98]. Similarly, Apligraf^®^ is made of cultured fibroblasts in a bovine collagen matrix, topped with cultured keratinocytes. But since these are human foreskin-derived neonatal cells, which are allogenic, they will eventually be rejected, limiting Apligraf^®^’s use to chronic wound healing [104]. Recent studies have also explored full skin substitutes using autologous cells. While promising, these techniques face significant challenges, such as lengthy culture times, high costs, and the potential absence of pigment cells. Regulatory hurdles further complicate their application, limiting their practical use in clinical settings [99,105].

#### 3.3.4. Cell-Based Therapies

Recent advancements in burn wound management highlight the effectiveness of integrating cellular therapies and dermal substitutes to enhance healing and improve aesthetic outcomes.

Studies have underscored the importance of cultured epidermal cells in burn treatment. Cultured epithelial autografts allow for the rapid expansion of a patient’s skin cells, offering a valuable solution for patients with extensive burns. This technique has several disadvantages, such as the fragility of keratinocyte layers, which makes them challenging to handle, high graft failure rates on areas like the lower back and buttocks, extended hospitalization, susceptibility to infection, and higher costs [106]. Since keratinocytes alone do not replace the dermis, additional methods must be associated. Studies show variability in the survival of cultured keratinocyte grafts, with improved outcomes when autografts are combined with cultivated grafts—achieving graft adherence rates between 73% and 96% [107,108].

Epicel^®^, a cultured epidermal autograft (CEA), is produced from human keratinocytes grown on a fibrin mesh scaffold. Despite its effectiveness, Epicel^®^’s use is restricted to extensive full-thickness burns covering over 30% of the body surface area due to its high costs, fragile nature, infection susceptibility, and demanding postoperative care requirements. Typically, it is combined with a permanent dermal substitute, although its practicality for dermatological applications is often limited by these same challenges [109].

A more recent alternative is a suspension of autologous keratinocytes, an FDA-approved device (ReCell^®^). This allows for the creation of a solution of the patient’s skin cells, harvested from a small skin biopsy, which is then sprayed over the burn wound. This method promotes epithelialization and accelerates wound closure without the need for extensive skin grafts. A biopsy of up to 4 cm^2^ can be turned into enough sprayable product to treat an area of 320 cm^2^ [110]. Clinical trials have shown that this approach significantly reduces healing time, improves pigmentation, and decreases scarring compared to traditional skin grafts [111].

Adipose-derived stem cells (ADSCs), which have a similar differentiation capacity to mesenchymal stem cells, applied on top of an ADM significantly accelerate post-burn wound reconstruction. ADSCs promote faster revascularization and cellular integration within the dermal matrix, yielding smoother skin texture and improved elasticity in treated areas, making it a promising approach for complex burn injuries. Adipose-derived stem cells (ADSCs) are particularly promising due to their availability from accessible tissue sources, through minimally invasive liposuction, or directly from the eschar. This ease of access and regenerative cell density makes ADSCs advantageous for wound healing and tissue repair applications [112,113,114].

While challenges remain, such as prolonged preparation times, these approaches are becoming integral in managing severe burns, particularly for cases where donor skin is limited. These cellular therapies continue to shape the future of reconstructive burn care by enabling personalized and biologically compatible treatments.

#### 3.3.5. Negative Pressure Wound Therapy

Negative pressure wound therapy (NPWT), also known as vacuum-assisted closure (VAC), accelerates wound healing by promoting vascularization, granulation, and faster re-epithelialization, while also reducing bacterial infections and edema in wounds. NPWT has been studied as a dressing for acute burn care, an intermediate treatment before grafting, an adjunct for autografts, a support for dermal substitutes, and a dressing for donor sites [115,116,117].

A study by Gümüş et al. demonstrated the efficacy of NPWT in managing high-voltage electrical burns, where traditional treatments often fail—an observation which was supported by subsequent studies from other centers [118,119]. Studies show that NPWT applied on autografts can lead to graft integration rates of up to 97% [116]. When used with ADMs, NPWT significantly improves outcomes, achieving a 96% success rate in dermal substitute integration with revascularization within one week, compared to the usual 2–3 weeks [120].

Table 2 showcases a summary of the therapeutic management of burn wounds throughout the three phases of evolution, each with its own specific particularities. It is important not to forget about pain management in this whole process, with drugs such as ibuprofen, acetaminophen or even opioids oftentimes being necessary.

## 4. Novel Approaches and Future Directions

Recent advancements in therapeutic approaches have significantly expanded the possibilities for reconstructive surgery, particularly for patients with complex soft tissue injuries or defects. Tissue bioengineering has emerged as a groundbreaking field, enabling the creation of customized solutions for regenerating and restoring soft tissues. Furthermore, the development of vascularized composite allotransplantation (VCA) represents a major leap forward, allowing for the transplantation of composite tissues—such as skin, muscle, bone, and nerves—in a single, integrated graft. This approach offers patients the possibility of full-scale tissue restoration, even in the most intricate and challenging cases. Moreover, the incorporation of nanotechnology into reconstructive medicine holds immense promise, with nanomaterials and nanoparticles being explored for their ability to enhance wound healing, improve tissue regeneration, and provide targeted drug delivery. Introducing these cutting-edge strategies into clinical practice is essential for providing comprehensive care that optimizes outcomes in terms of structural integrity, function, and aesthetics. By leveraging these innovations, healthcare providers can deliver more effective treatments, which not only restore patients’ physical appearance but also significantly enhance their quality of life. The ultimate goal is to help patients regain their pre-injury functionality as quickly as possible, supporting both their social reintegration and professional rehabilitation. These advancements mark a shift toward more personalized and precise reconstructive care, enabling patients to not only recover their health but also regain their confidence, independence, and sense of self [130,131,132].

### 4.1. Bioprinting Technologies

Current skin substitutes have certain disadvantages, among which is the incapacity to stimulate the regeneration of vessels, nerves, sweat glands and sebaceous glands, hair follicles, or pigmentation. Bioprinting can innovate the field by generating complex skin constructs that include all natural skin structures, significantly improving the outcome of burn patients [133,134,135].

Bioprinting technology represents a groundbreaking advancement in burn wound therapy. This technique involves the layer-by-layer deposition of living cells with hydrogel-based scaffolds (“bioink”) to create complex tissues, such as skin, vascular grafts, heart tissue, or cartilaginous structures. Bioprinting allows for the fabrication of skin grafts tailored to specific defects, which may reduce dependence on donor sites and enhance graft integration [133,136].

Tridimensional bioprinting consists of five steps: scanning the target tissue, developing a model based on the imaging data using computer-aided design and manufacturing software, selecting appropriate biomaterial scaffolds and cell types, printing the tissue using a bioprinter, and, lastly, allowing the printed tissue to mature [133].

Bioprinting has several advantages for developing skin constructs over conventional tissue engineering methods, such as automation, standardization for clinical scenarios, and precise cell placement. While traditional tissue engineering approaches, such as culturing cells on a scaffold and maturing them in a bioreactor, have comparable results to bioprinting, there are still challenges to address in producing skin constructs. Notably, conventional methods often involve lengthy production times, especially when needed for extensive burns [137].

There are two main bioprinting approaches: in situ and in vitro. In situ bioprinting involves the precise deposition of cells on the wound bed, enabling skin maturation directly at the wound site, without the need for the costly and time-consuming in vitro differentiation or multiple surgeries [138]. On the other hand, in vitro bioprinting involves skin maturation in a bioreactor and then transplanting the construct onto the wound site. Studies have shown the effectiveness of both methods, with in situ bioprinting showing promising results in wound healing and skin regeneration, including successful trials in animal models [133].

Recent studies have shown that bioprinted skin constructs can replicate the multi-layered structure of human skin, including both dermal and epidermal components. The epidermis is thin and can potentially be bioprinted using laser-assisted technology to replicate its morphology and pigmentation. The basement membrane is composed of fibrous tissue and ECM components, but bioprinting it is challenging, so researchers often rely on tissue self-assembly. The dermis, found beneath the basement membrane, contains fibroblasts, ECM, skin appendages, blood vessels, and nerves. This layer’s elasticity and strength come from collagen and elastin fibers. Extrusion-based bioprinting could be effective for this layer, allowing the inclusion of various cell types like hair follicles and glands. The hypodermis, below the dermis, consists of adipose tissue that provides insulation and padding, and restoring it through autologous fat injection has shown benefits in burn recovery by improving scar pliability. This technique has the potential to produce skin grafts with improved vascularization, reducing the likelihood of graft failure. Moreover, there are studies looking into the possibility of incorporating other structures into the skin constructs, such as melanocytes, hair follicles, endothelial cells, sweat glands, and sebaceous glands [133,134,135].

To advance the clinical use of bioprinting for skin, several technological challenges must be addressed. First, large quantities of cells are needed for transplant-ready skin, and current cell expansion methods only support millions, not billions of cells, necessitating innovation in cell expansion technologies. Additionally, improvements in bioinks are required to ensure consistent bioprinting with appropriate biomechanical properties. Enhanced printing resolution is crucial to replicate the microarchitecture of skin, and control over the microarchitecture will be vital for functional tissue. Increasing printing speed without compromising cell viability is another challenge. Building a functional vasculature is also essential for tissue perfusion, and methods using sacrificial inks or simultaneous printing of vasculature and surrounding cells show promise. Additionally, the development of standard growth media, ECM-based bioinks, and dynamic bioreactors will improve tissue maturation. Future efforts should focus on refining computational and analytical approaches to model and optimize bioprinting parameters and tissue development [133].

### 4.2. Wireless Microcurrent Stimulation

Wireless Microcurrent Stimulation (WMCS) is an emerging technique that uses electrical currents to stimulate cell activity, including DNA synthesis, cell migration, and collagen production. This non-invasive therapy, which delivers a current to the burn wound without direct contact, generates a significant number of oxygen molecules, which can emit electrons at the time of contact with the wound [139]. This repetitive interaction generates a constant low-intensity electric current, improving blood flow in burn wounds without any systemic adverse effects or impairing wound healing. These electric currents mimic the natural current that the body generates for tissue repair [140].

WMCS enhances wound healing, especially by reducing the zone of stasis, and reduces pain in burn patients [139]. An important aspect that needs to be considered is the compatibility of this technique with specific patient characteristics, since those with cardiac pacemakers or metal implants cannot be treated with WMCS. A study comparing NPWT and WMCS proved that WMCS has superior results when considering the reduction of the burn wound area, while NPWT is better for the reduction of bacterial growth, although other studies suggest that WMCS has bacteriostatic effects as well [140].

### 4.3. Extracorporeal Shock Wave Therapy

Extracorporeal Shock Wave Therapy (ESWT) has been explored as a novel approach to enhance burn wound healing, due to its safety, efficiency, non-invasiveness, and lower costs. It uses electromagnetic shock wave energy with several biologic effects, such as increased cellular activity and tissular regeneration, increased blood flow, and reduced inflammation [141,142].

Studies show that ESWT may increase wound perfusion and reduce the need for grafting in deep partial-thickness and full-thickness burns [143,144]. When used for superficial partial-thickness burns, ESWT has shown significantly faster re-epithelization times after debridement or topical therapy, in a prospective randomized phase II trial [145]. Similarly, the donor site for the split-thickness partial graft can be managed with a single round of ESWT immediately after harvest, significantly accelerating donor site healing [146]. Several studies investigated the effects of ESWT on pain, pruritus, quality of life, burn scars, and their microbiome, with significantly reduced scar pain, positive influences on the skin’s supportive microbiome, and improved healing outcomes in burn patients [147,148,149].

### 4.4. Nanotechnology and Nanomedicine

Nanotechnology is rapidly advancing in the field of burn wound care, offering new methods for preventing infection and promoting healing [150,151]. Nanoparticles are being investigated not only for regeneration and repair, but also for their ability to deliver therapeutic agents directly to the wound site, improving the efficacy of treatment while minimizing systemic side effects. Early research indicates that nanotechnology can enhance collagen deposition, hair follicle regeneration, and overall wound healing [152,153].

Organic nanostructures include polymeric nanoparticles, nanoemulsions, nanogels, liposomes, solid lipid-based nanoparticles, etc. On the other hand, nanostructures can also be inorganic, based on nanocarbons (such as carbon nanotubes, graphene, or nanodiamonds), gold, copper, silver, titanium dioxide, magnetic nanoparticles, or quantum dots [150]. These act as delivery methods for different agents that need to be locally administered on the wound, such as antibiotics, growth factors, specific genes, or even stem cells, preventing wound infection whilst stimulating tissue regeneration [151,154]. Nanotechnology can also be used to create nanoengineered scaffolds based on nanoparticles and nanofibers in order to provide enhanced wound protection, achieve lower wound infections, and accelerate healing through increased cell attachment [151,155].

However, the potential toxicity of metal-based nanoparticles remains a significant concern. Studies indicate that certain nanoparticles, such as silver and titanium dioxide, may generate reactive oxygen species or accumulate in tissues, causing cytotoxicity, oxidative stress, or inflammatory responses. These effects can hinder wound healing and pose risks for long-term systemic exposure. Addressing this challenge requires optimizing nanoparticle formulations to balance therapeutic benefits with biocompatibility. Strategies such as surface functionalization or incorporating biodegradable coatings are under investigation to mitigate toxicity while maintaining efficacy [156,157].

### 4.5. Vascularized Composite Allotransplantation (VCA)

Vascularized composite allografts have been carried out for life-enhancing indications in a carefully chosen group of patients, in accordance with specific institutional protocols. After two decades of clinical research on vascularized composite grafts, the primary limitation remains the need for lifelong immunosuppression, even with standardized indications and surgical protocols, for what is ultimately a non-vital procedure. This prolonged immunosuppressive treatment is associated with a range of adverse reactions. At present, the focus of composite tissue transplant research lies in the field of immunology [158,159,160].

VCA has been successfully used for burn reconstruction since 2003, providing a solution for patients with severe deformities. Traditional reconstructive methods following extensive facial burns often result in suboptimal functional and aesthetic outcomes. The reconstruction of burn injuries affecting central facial units or uniquely intricate structures, such as the eyelids, remains particularly challenging [161,162].

However, its safety in this population has been a concern due to the high levels of alloimmunization resulting from tissue exposure during burn care. Pre-VCA factors, such as multiple transfusions, prior transplants, pregnancies, the use of skin allografts, and mechanical support devices, can lead to sensitization. This may ultimately exclude burn patients—those who could benefit the most from VCA—from receiving hand or face transplants [161,163].

Vascularized composite allotransplantation (VCA) for burn reconstruction has been associated with an increased risk of 1-year mortality and an increased number of episodes of acute rejection compared to other VCA-candidate patients. Future research should aim to identify the unique risk factors specific to burn patients undergoing VCA and explore the relationship between antigenic burdens and surgical outcomes. Although burn patients currently represent a minority of VCA recipients, they constitute the largest demographic on waiting lists due to the high burden of donor-specific antibodies, which complicates donor matching [161,163].

The current goal is to reduce immunosuppression to a level where both the functionality of the allograft is preserved, and adverse immune reactions are minimized. The long-term objective is to achieve donor-specific immune tolerance, where the immune system no longer reacts to the donor’s antigens, eliminating the need for ongoing immunosuppressant therapy [159,164].

## 5. Conclusions

Burn wound therapy is experiencing a major transformation with the integration of advanced technologies and novel therapeutic strategies. Cutting-edge techniques are enhancing patient outcomes by promoting faster and more effective wound healing, lowering infection risks, and minimizing scar formation. While these innovations show substantial promise, several challenges remain in their implementation within clinical settings. These include the need for standardized protocols, high costs, and the requirement for specialized equipment and training. Moreover, the variability in patient conditions, wound types, and response to treatment further complicates the widespread adoption of these methods. Continued research and clinical trials are crucial to further refine these methods, optimize their effectiveness, and make them more accessible to a broader range of patients. As these technologies advance, the future of burn care could bring dramatic improvements in both the functional recovery and aesthetic appearance of healed burn wounds, ultimately enhancing the quality of life for patients.

## Figures and Tables

**Figure 5:**
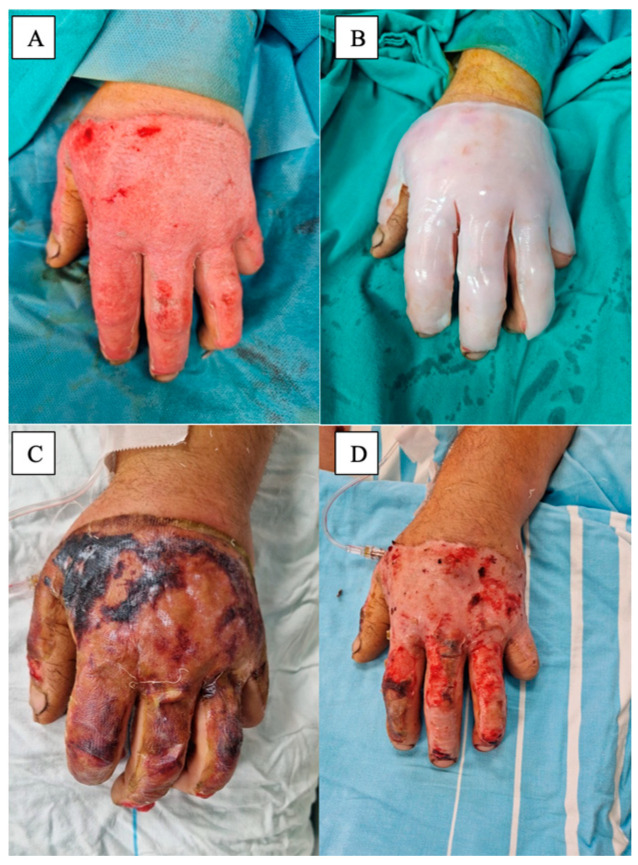
(**A**) Partial-thickness burn on the dorsum of the hand. (**B**) Epicite Hydro^®^ placed on the burn wound. (**C**) Aspect of the dressing providing wound coverage during treatment. (**D**) Final result, with almost complete re-epithelization.

**Table 1:** Classification of temporary skin substitutes used in burn treatment and their characteristics [93,94,95,96,97].

Synthetic Skin Substitutes [93,94,95,96,97]				
Dressing Type	Action Mechanism and Advantages	Indication	Disadvantages	Examples
Alginate	Forms a gel when in contact with exudate, promoting a moist wound healing environment. Highly absorbent.	Used for moderate to severe partial-thickness burns (second-degree burns) with exudation; helps in managing fluid loss.	Requires secondary dressing; not suitable for dry wounds.	Kaltostat^®^, Algisite^®^, Sorbsan^®^, Sorbalgon^®^, Algicell^®^.
Film	Thin, transparent, and adhesive. Provides a moisture barrier while allowing oxygen and vapor exchange.	Ideal for superficial burns (first-degree burns) or minor burns; provides moisture protection with minimal exudate.	Not absorbent; may cause maceration in highly exudative burns.	Opsite^®^, Tegaderm^®^, Bioclusive^®^, Mepore^®^.
Hydrocolloid	Forms a gel upon contact with exudate, which helps in autolytic debridement. Provides moisture and thermal insulation.	Effective for superficial to moderate partial-thickness burns (second-degree); promotes healing with minimal exudation and reduces pain.	Can cause maceration in highly exudative burns; not suitable for infected wounds.	Duoderm^®^, Comfeel^®^, Hydrocoll^®^, Gentell Dermatell^®^, Epicite Hydro^®^ (see Figure 5).
Hydrogel	Provides hydration and cooling for dry wounds, promoting autolytic debridement. Can also reduce pain and friction.	Best for dry partial-thickness burns, necrotic burns, or deep burns that require hydration and pain relief.	Not suitable for highly exudative burns; may require secondary dressing.	Intrasite^®^, Aquacel Hydrogel^®^, Solcoseryl^®^, Dermaplex^®^, Dermagran^®^.
Foams	Absorbent and provide cushioning. Maintain a moist wound environment and are typically non-adherent.	Used for moderate to severe exudative burns (second-degree burns) and deep partial-thickness burns with significant exudate.	May require frequent changes; not suitable for dry or shallow burns.	Allevyn^®^, Lyofoam^®^, Mepilex^®^, Hydrocell^®^.
Silicone	Gentle adhesion to the skin without damaging surrounding tissue. Reduces pain and trauma during dressing changes.	Suitable for burns requiring scar management, including burn scars, sensitive skin, or skin grafts.	Expensive; not suitable for highly exudative wounds.	Mepitel^®^, Silgel^®^, Hypafix^®^, Mepiform^®^, Suprathel^®^.
Silver	Antimicrobial properties due to the release of silver ions, which inhibit bacterial growth.	Indicated for infected burns or high-risk burn wounds (e.g., deep partial-thickness and full-thickness burns) prone to infection.	Prolonged use may lead to silver toxicity; expensive.	Acticoat^®^, Aquacel Ag^®^, Silvercel^®^, Atrauman Ag^®^.

**Table 2:** Therapeutic management of burn wounds in a dynamic evolutive setting [85,121,122,123,124,125,126,127,128,129].

Dynamic Therapeutic Management of Burn Wounds [85,121,122,123,124,125,126,127,128,129]			
Burn Wound Depth	First 24–72 h	72 h–3 Weeks	Scar Remodeling
Second degree	Silver sulfadiazine Antimicrobial and antiseptic topical agents Non-adherent dressings Pain management	Temporary coverage: allografts, xenografts, HAM Hydrocolloid dressings Hydrogel dressings Alginate dressings Foams Silver dressings NPWT Non-adherent dressings Pain management	Silicone sheets or gels Emollient creams Vitamin E ointments Compression garments Retinoids
Third degree		Early excision and coverage Temporary coverage: allografts, xenografts, HAM Permanent coverage: NPWT + skin grafts, NPWT + allograft + meshed skin graft *On grafted areas:* Cultured epidermal autografts ADMs Foams Silver dressings Non-adherent dressings *On donor areas:* Film dressings Foam dressings Alginate dressings Ointments, sprays Pro-epithelization agents	
Infected wounds	Serial debridement Antiseptic agents and dressings (silver dressings) Instillation systems Topical antimicrobials according to the antibiogram results		

## Data Availability

This paper is a literature review; no new data were created.
